# A Low-Dimensional Radial Silhouette-Based Feature for Fast Human Action Recognition Fusing Multiple Views

**DOI:** 10.1155/2014/547069

**Published:** 2014-10-29

**Authors:** Alexandros Andre Chaaraoui, Francisco Flórez-Revuelta

**Affiliations:** ^1^Department of Computer Technology, University of Alicante, P.O. Box 99, 03080 Alicante, Spain; ^2^Faculty of Science, Engineering and Computing, Kingston University, Penrhyn Road, Kingston upon Thames KT1 2EE, UK

## Abstract

This paper presents a novel silhouette-based feature for vision-based human action recognition, which relies on the contour of the silhouette and a radial scheme. Its low-dimensionality and ease of extraction result in an outstanding proficiency for real-time scenarios. This feature is used in a learning algorithm that by means of model fusion of multiple camera streams builds a bag of key poses, which serves as a dictionary of known poses and allows converting the training sequences into sequences of key poses. These are used in order to perform action recognition by means of a sequence matching algorithm. Experimentation on three different datasets returns high and stable recognition rates. To the best of our knowledge, this paper presents the highest results so far on the MuHAVi-MAS dataset. Real-time suitability is given, since the method easily performs above video frequency. Therefore, the related requirements that applications as ambient-assisted living services impose are successfully fulfilled.

## 1. Introduction

Human action recognition has been in great demand in the field of pattern recognition, given its direct relation to video surveillance, human-computer interaction, and ambient-assisted living (AAL), among other application scenarios. Especially in the latter, human behavior analysis (HBA), in which human action recognition plays a fundamental role, can endow smart home services with the required “smartness” needed to perform fall detection, intelligent safety services (closing a door or an open tap), or activities of daily living (ADL) recognition. Upon these detection stages, AAL services may learn subjects' routines, diets, and even personal hygiene habits, which allow providing useful and proactive services. For this reason, human action recognition techniques are essential in order to develop AAL services that support safety at home, health assistance, and aging in place.

Great advances have been made in vision-based motion capture and analysis [1], and, at the same time, the society is starting to demand sophisticated and accurate HBA systems. This is shown, for example, in the recent rise of interest in devices like the Microsoft Kinect sensor. Current efforts focus on achieving admissible recognition speeds [2], which is essential for real-time and online systems. Another goal is the successful handling of multiview scenarios so as to add robustness to occlusions and improve the quality of the recognition [3]. One of the main drawbacks of multiview techniques is that rich and detailed 3D scene reconstructions are normally incompatible with real-time recognition. On the other hand, simpler 2D-based methods fail to achieve the same recognition robustness [4].

The current proposal builds upon earlier work, where we have presented a human action recognition method based on silhouettes and sequences of key poses, which shows to be suitable for real-time scenarios and specially robust to actor variances [5]. In [6], a study is included comparing different approaches of fusing multiple views using an approach based on a bag of key poses, which is extended in the present contribution. One method stage in which substantial computational cost is added and success in later stages depends upon is feature extraction. In this paper, this specific stage is especially targeted. A low-dimensional radial silhouette-based feature is combined with a simple learning approach based on multiple video streams. Working with silhouette contour points, radial bins are computed using the centroid as the origin, and a summary representation is obtained for each bin. This pose representation is used in order to obtain the per-view key poses which are involved in each action performance. Therefore, a model fusion of multiple visual sensors is applied. From the obtained bag of key poses, the sequences of key poses of each action class are computed, which are used later on for sequence matching and recognition. Experimentation performed on two publicly available datasets (Weizmann [7] and MuHAVi [8]) and a self-recorded one shows that the proposed technique not only obtains very high and stable recognition rates but also proves to be suitable for real-time applications. Note that by “real time” we mean that recognition can be performed at video frequency or above, as is common in the field.

The remainder of this paper is organized as follows.  Section 2 summarizes the most recent and relevant works in human action recognition focusing on the type of features used and how multiview scenarios are managed. In Section 3, our proposal is detailed offering a low-dimensional feature based on silhouette contours and a radial scheme. Sections 4 and 5 specify the applied multiview learning approach based on a bag of key poses and action recognition through sequence matching.  Section 6 analyzes the obtained results and compares them with the state of the art in terms of recognition rate and speed, providing also an analysis of the behaviour of the proposed method with respect to its parameters. Finally, we present conclusions and discussion in Section 7.

## 2. Related Work

### 2.1. Feature Extraction

Regarding the feature extraction stage of human action recognition methods based on vision, these can be differentiated by the either static or dynamic nature of the feature. Whereas static features consider only the current frame (extracting diverse types of characteristics based on shape, gradients, key points, etc.), dynamic features consider a sequence of several frames and apply techniques like image differencing, optical flow, and spatial-temporal interest points (STIP).

Among the former, we find silhouette-based features which rely either on the whole shape of the silhouette or only on the contour points. In [19], action primitives are extracted reducing the dimensionality of the binary images with principal component analysis (PCA). Polar coordinates are considered in [20], where three radial histograms are defined for the upper part, the lower part, and the whole human body. Each polar coordinate system has several bins with different radii and angles, and the concatenated normalized histograms are used to describe the human posture. Similarly, in [21], a log-polar histogram is computed choosing the different radii of the bins based on logarithmic scale. Silhouette contours are employed in [10] with the purpose of creating a distance signal based on the pointwise Euclidean distances between each contour point and the centroid of the silhouette. Conversely, in [22], the pairwise distances between contour points are computed to build a histogram of distances resulting in a rotation, scale, and translation invariant feature. In [9], the whole silhouette is used for gait recognition. An angular transform based on the average distance between the silhouette points and the centroid is obtained for each circular sector. This shows robustness to segmentation errors. Similarly, in [23], the shape of the silhouette contour is projected on a line based on the *ℜ* transform, which is then made invariant to translation. Silhouettes can also be used to obtain stick figures, for instance, by means of skeletonization. Chen et al. [24] applied star skeletonization to obtain a five-dimensional vector in star fashion considering the head, the arms, and the legs as local maxima. Pointwise distances between contour points and the centroid of the silhouette are used to find the five local maxima. In the work of İkizler and Duygulu [11], a different approach based on a “bag-of-rectangles” is presented. In their proposal, oriented rectangular patches are extracted over the human silhouette, and the human pose is represented with a histogram of circular bins of 15° each.

A very popular dynamic feature in pattern recognition based on computer vision is optical flow. Fathi and Mori [13] rely on low-level features based on optical flow. In their work, weighted combinations of midlevel motion features are built covering small spatiotemporal cuboids from which the low-level features are chosen. In [25], motion over a sequence of frames is considered defining motion history and energy images. These encode the temporal evolution and the location of the motion, respectively, over a number of frames. This work has been extended by [26] so as to obtain a free-viewpoint representation from multiple views. A similar objective is pursued in [7], where time is considered as the third dimension building space-time volumes based on sequences of binary silhouettes. Action recognition is performed with global space-time features composed of the weighted moments of local space-time saliency and orientation. Cherla et al. [27] combine eigenprojections of the width profile of the actor with the centroid of the silhouette and the standard deviation in the *X* and *Y* axes in a single feature vector. Robustness to occlusions and viewpoint changes is targeted in [28]. A 3D histogram of oriented gradients (3DHOG) is computed for densely distributed regions and combined with temporal embedding to represent an entire video sequence. Tran and Sorokin [12] merge both silhouette shape and optical flow in a 286-dimensional feature, which also includes the context of 15 surrounding frames reduced by means of PCA. This feature has been used successfully in other works as, for instance, recently in [29]. Rahman et al. [30] take an interesting approach proposing a novel feature extraction technique, which relies on the surrounding regions of the subjects. These negative spaces present advantages related to robustness to boundary variations caused by partial occlusions, shadows, and nonrigid deformations.

RGB-D data, that is, RGB color information along pixel-wise depth measurement, is increasingly being used, since the Microsoft Kinect device has been released. Using the depth data and relying on an intermediate body part recognition process, a markerless body pose estimation in form of 3D skeletal information can be obtained in real time [2]. This kind of data results proficient for gesture and action recognition required by applications, such as gaming and natural user interfaces (NUI) [31]. In [31, 32], more detailed surveys about these recently appeared depth-based methods can be found.

Naturally, the usage of static features does not mean that the temporal aspect cannot be considered. Temporal cues are commonly reflected in the change between successive elements of a sequence of features or in the learning algorithm itself. For further details about the state of the art, we refer to [1, 33].

### 2.2. Multiview Recognition

Another relevant area for this work is how human action recognition is handled when dealing with multiple camera views. Multiview recognition methods can be classified, for example, by the level at which the fusion of information happens. Initially, when dealing with 2D data from multiple sources, these can be used in order to create a 3D representation [34, 35]. This data fusion allows applying a single feature extraction process which minimizes information loss. Nevertheless, 3D representations usually imply a higher computational cost as appropriate 3D features need to be obtained. Feature fusion places the fusion process one step further by obtaining single-view features for each of the camera views and generating a common representation for all the features afterwards. The fusion process depends on the type of data. Feature vectors are commonly combined by aggregation functions or concatenation of vectors [36, 37] or also more sophisticated techniques as canonical correlation analysis [29]. The appeal of this type of fusion is the resulting simplicity of transition from single- to multiview recognition methods, since multiview data is only handled implicitly. A learning method which in fact learns and extracts information from actions or poses from multiple views requires considerations at the learning scheme. Through model fusion, multiple views are learned either as other possible instances of the same class [36] or by explicitly modelling each possible view [38]. These 2D or 3D models may support a limited or unlimited number of points of view (POV). Last but not least, information fusion can be applied at the decision level. In this case, for each of the views, a single-view recognition method is used independently, and a decision is taken based on the single-view recognition results. The best view is chosen based on one or multiple criteria like closest distance to the learned pattern, highest score/probability of feature matching, or metrics which try to estimate the quality of the received input pattern. However, the main difficulty is to establish this decision rule because it depends strongly on the type of actions to recognize and on the camera setup. For example, in [39], a local segment similarity voting scheme is employed to fuse multiple views, and superior results are obtained when compared with feature fusion based on feature concatenation. Finally, feature extraction and fusion of multiple views do not necessarily have to be considered two separate processing stages. For instance, in [40, 41], lattice computing is proposed for low-dimensional representation of 2D shapes and data fusion.

In our case, model fusion has been chosen because of two reasons: (1) in comparison with fusion at the decision level, only a single learning process is required in order to perform multiview recognition and (2) it allows explicit modeling of the poses from each view that are involved in a performance of an action. As a consequence, multiple benefits can be obtained as follows.

1. Once the learning process has been finished, further views and action classes can be learned without restarting the whole process. This leads to supporting incremental learning and eliminating the widely accepted limitation of batch-mode training for human action recognition [42].
2. The camera setups do not need to match between training and testing stages. More camera views may improve the result of the recognition, though it is not required to have all the cameras available.
3. Each camera view is processed separately and matched with the corresponding view, without requiring to know specifically at which angle it is installed.

These considerations are important requirements in order to apply the proposed method to the development of AAL services. Model fusion enabled us to fulfil these constraints, as will be seen in the following sections.

## 3. Pose Representation Feature

As has been previously introduced, our goal is to perform human action recognition in real time and to do so even in scenarios with multiple cameras. Therefore, the computational cost of feature extraction needs to be minimal. This leads us to the usage of silhouette contours. Human silhouettes contain rich shape information and can be extracted relatively easily, for example, through background subtraction or human body detection. In addition, silhouettes and their contours show certain robustness to lighting changes and small viewpoint variations compared to other techniques, as optical flow [43]. Using only the contour points of the silhouette results in a significant dimensionality reduction by getting rid of the redundant interior points.

The following variables are used along this section:

(1) the number of contour points *n*;
(2) the number of radial bins *S*;
(3) the indices *i*, *j*, *k*, and *l*, for all *i*, *k*, *l* ∈ {1,…, *n*} and for all *j* ∈ {1,…, *S*}.

**Figure 1:**
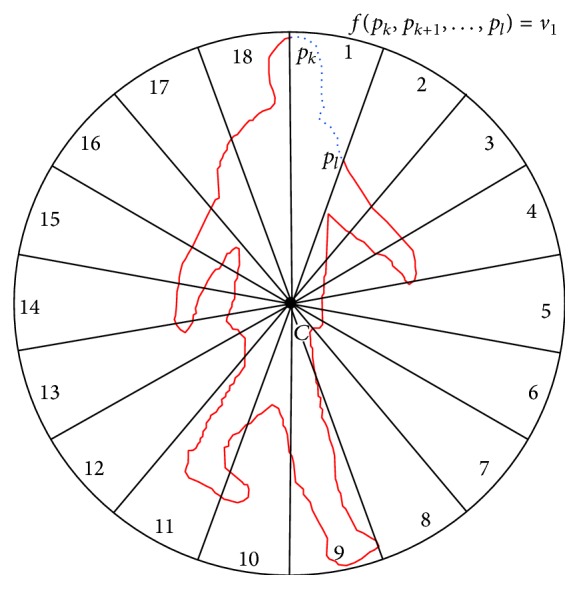
Overview of the feature extraction process. (1) All the contour points are assigned to the corresponding radial bin; (2) for each bin, a summary representation is obtained. (Example with 18 bins.)

We use the border following algorithm from [44] to extract the *n* contour points **P** = {*p*_1_, *p*_2_,…, *p*_*n*_}, where *p*_*i*_ = (*x*_*i*_, *y*_*i*_). Our proposal consists in dividing the silhouette contour in *S* radial bins of the same angle. Taking the centroid of the silhouette as the origin, the specific bin of each contour point can be assigned. Then, in difference to [20, 21] where radial or log-polar histograms are used as spatial descriptors or [24] where star skeletonization is applied, in our approach, an efficient summary representation is obtained for each of the bins, whose concatenation returns the final feature (Figure 1 shows an overview of the process).

The motivation behind using a radial scheme is two-fold. On one hand, it relies on the fact that when using a direct comparison of contours, even after length normalization as in [10], spatial alignment between feature patterns is still missing. Each silhouette has a distinct shape depending on the actor and the action class, and therefore a specific part of the contour can have more or less points in each sample. Using an element-wise comparison of the radial bins of different contours, we ignore how many points each sample has in each bin. This avoids an element-wise comparison of the contour points, which would imply the erroneous assumption that these are correlated. On the other hand, this radial scheme allows us to apply an even further dimensionality reduction by obtaining a representative summary value for each radial bin.

The following steps are taken to compute the feature.

(1) The centroid of the contour points *C* = (*x*_*c*_, *y*_*c*_) is calculated as
  (1) $$\begin{array}{c} x_{c}=\frac{\sum_{i=1}^{n}x_{i}}{n},\;\;y_{c}=\frac{\sum_{i=1}^{n}y_{i}}{n}. \end{array}$$
(2) The pointwise Euclidean distances between each contour point and the centroid, **D** = {*d*_1_, *d*_2_,…, *d*_*n*_}, are obtained as in [10]. Consider
  (2) $$\begin{array}{c} d_{i}=||C_{m}-p_{i}||,\;\forall i\in \left\{1,\ldots ,n\right\}. \end{array}$$
(3) Considering a clockwise order, the corresponding bin *s*_*i*_ of each contour point *p*_*i*_ is assigned as follows (for the sake of simplicity, *α*_*i*_ = 0 is considered as *α*_*i*_ = 360):
  (3) $$\begin{array}{c} \alpha_{i}=\left\{\begin{array}{cc} \text{arccos}\left(\frac{y_{i}-y_{c}}{d_{i}}\right)\cdot \frac{180}{\pi }, & \text{if}\;\;x_{\text{i}}\geq 0,\\ 180+\text{arccos}\left(\frac{y_{i}-y_{c}}{d_{i}}\right)\cdot \frac{180}{\pi }, & \text{otherwise}, \end{array}\right.\\ s_{i}=\left\lceil \frac{S\cdot \alpha_{i}}{360}\right\rceil ,\;\forall i\in \left\{1,\ldots ,n\right\}. \end{array}$$
(4) Finally, a summary representation is obtained for the points of each bin. The final feature $\bar{V}$ results of the concatenation of summary representations. These are normalized to unit sum in order to achieve scale invariance:
  (4) vj=fpk,pk+1,…,plsk,…,sl=j∧k, l∈1,…,n,kkkkkkkkkkkkkkkkkkkkkkkkkkk∀j∈1,…,S,vj¯=vj∑0=1Sv0, ∀j∈1,…,S,V¯=v1¯||v2−||···||vS¯.

The function *f* could be any type of function which returns a significant value or property of the input points. We tested three types of summaries (variance, max value, and range), based on the previously obtained distances to the centroid, whose results will be analyzed in Section 6.

The following definitions of *f* are used:

(5) $$\begin{array}{c} f_{\text{var}}\left(p_{k},p_{k+1},\ldots ,p_{l}\right)=\overset{l}{\underset{i=k}{\sum }}{\left(d_{i}-\mu \right)}^{2}, \end{array}$$

where *μ* is the average distance of the contour points of each bin. Consider

(6) fmax⁡pk,pk+1,…,pl=max⁡dk,dk+1,…,dl,frangepk,pk+1,…,pl=max⁡dk,dk+1,…,dl −min⁡dk,dk+1,…,dl.

**Figure 2:**
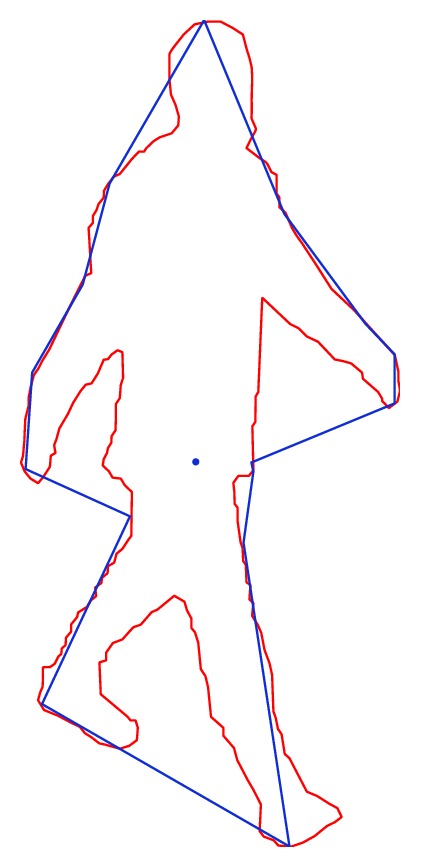
Example of the result of applying the *f*_max⁡_ summary function.

Figure 2 shows an example of the result of the *f*_max⁡_ summary function.

## 4. Multiview Learning Algorithm

Considering that multiple views of the same field of view are available, our method learns from these views at the model level, relying therefore on model fusion.* K-*means clustering is used in order to identify the per-view representative instances, the so-called key poses, of each action class. The resulting bag of key poses serves as a dictionary of known poses and can be used to simplify the training sequences of pose representations to sequences of key poses.

**Figure 3:**
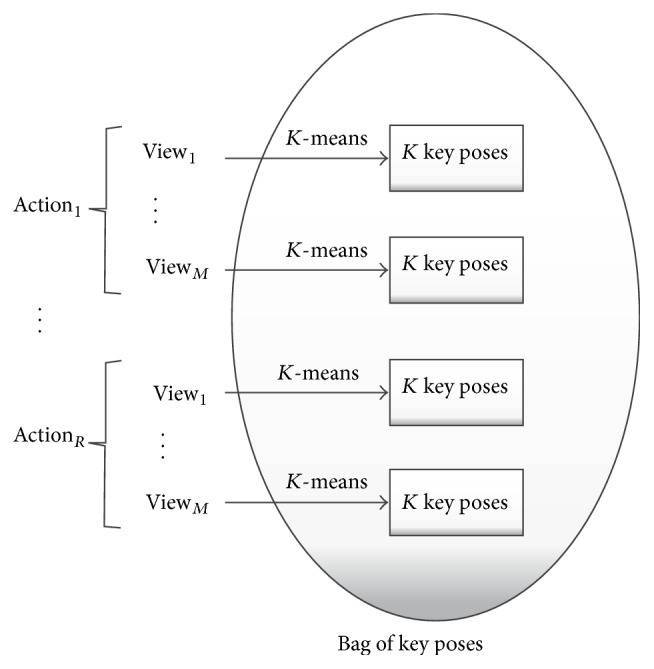
Overview of the generation process of the bag of key poses. For each action, the per-view key poses are obtained through* K-*means clustering, taking the cluster centers as representatives.

First, all the training video sequences need to be processed to obtain their pose representations. Supposing that *M* views are available and *R* action classes need to be learned,* K-*means clustering with Euclidean distance is applied for the pose representations of each combination of view and action class separately. Hence, *K* clusters are obtained for each of the *M* × *R* groups of data. The center of each cluster is taken as a key pose, and a bag of key poses of *K* × *M* × *R* class representatives is generated. In this way, an equal representation of each of the action classes and fused views can be assured in the bag of key poses (Figure 3 shows an overview of the process).

At this point, the training data has been reduced to a representative model of the key poses that are involved in each view of each action class. Nevertheless, not all the key poses are equally important. Very common poses such as standing still are not able to distinguish between actions, whereas a* bend* pose can most certainly be only found in its own action class. For this reason, a weight *w* which indicates the capacity of discrimination of each key pose *kp* is obtained. For this purpose, all available pose representations are matched with their nearest neighbor among the bag of key poses (using Euclidean distance) so as to obtain the ratio of within-class matches *w*_*kp*_ = *matches*_*kp*_/*assignments*_*kp*_. In this manner,* matches* is defined as the number of within-class assignments, that is, the number of cases in which a pose representation is matched with a key pose from the same class, whereas* assignments* denotes the total number of times that key pose got chosen. Please see Algorithm 1 for greater detail.

Video recognition presents a clear advantage over image recognition which relies on the temporal dimension. The available training sequences present valuable information about the duration and the temporal evolution of action performances. In order to model the temporal relationship between key poses, the training sequences of pose representations are converted into sequences of key poses. For each sequence, the corresponding sequence of key poses Seq = {*kp*_1_, *kp*_2_,…, *kp*_*t*_} is obtained by interchanging each pose representation with its nearest neighbor key pose among the bag of key poses. This allows us to capture the long-term temporal evolution of key poses and, at the same time, to significantly improve the quality of the training sequences as noise and outliers are filtered.

## 5. Action Recognition

During the recognition stage, the goal is to assign an action class label to an unknown sequence. For this purpose, the video sequence is processed in the same way as the training sequences were. (1) The corresponding pose representation of each video frame is generated and (2) the pose representations are replaced with the nearest neighbor key poses among the bag of key poses. This way, a sequence of key poses is obtained and recognition can be performed by means of sequence matching.

Since action performances can nonuniformly vary in speed depending on the actor and his/her condition, sequences need to be aligned properly. Dynamic time warping (DTW) [45] shows proficiency in temporal alignment of sequences with inconsistent lengths, accelerations, or decelerations. We use DTW in order to find the nearest neighbor training sequence based on the lowest DTW distance.

Given two sequences Seq = {*kp*_1_, *kp*_2_,…, *kp*_*t*_} and Seq′ = {*kp*_1_′, *kp*_2_′,…, *kp*_*u*_′}, the DTW distance *d*_DTW_(Seq, Seq′) can be obtained as follows:

(7) $$\begin{array}{c} d_{\text{DTW}}\left(\text{Seq},{\text{Seq}}^{\text{'}}\right)=\text{dtw}\left(t,u\right),\\ \text{dtw}\left(i,j\right)=\min\left\{\begin{array}{c} \text{dtw}\left(i-1,j\right),\\ \text{dtw}\left(i,j-1\right),\\ \text{dtw}\left(i-1,j-1\right) \end{array}\right\}+d\left({kp}_{i},{kp}_{j}^{\text{'}}\right), \end{array}$$

where the distance between two key poses *d*(*kp*_*i*_, *kp*_*j*_′) is obtained based on both the Euclidean distance between their features and the relevance of the match of key poses. As seen before, not all the key poses are as relevant for the purpose of identifying the corresponding action class. Hence, it can be determined how relevant a specific match of key poses is based on their weights *w*_*i*_ and *w*_*j*_′.

In this sense, the distance between key poses is obtained as

(8) $$\begin{array}{c} d\left({kp}_{i},{kp}_{j}^{\text{'}}\right)=\left|{kp}_{i}-{kp}_{j}^{\text{'}}\right|+z\mathrm{rel}\left(i,j\right),\\ \mathrm{rel}\left(i,j\right)=\left|\text{dev}\left(i,j\right)*w_{i}*w_{j}^{\text{'}}\right|,\\ \text{dev}\left(i,j\right)=\left|{kp}_{i}-{kp}_{j}^{\text{'}}\right|-\text{average\_distance}, \end{array}$$

where average_distance corresponds to the average distance between key poses computed throughout the training stage. As it can be seen, the relevance *rel*⁡(*i*, *j*) of the match is determined based on the weights of the key poses, that is, the capacity of discrimination and the deviation of the feature distance. Consequently, matches of key poses which are very similar or very different are considered more relevant than those that present an average similarity. The value of *z* depends upon the desired behavior.  Table 1 shows the chosen value for each case. In pairings of discriminative key poses which are similar to each other, a negative value is chosen in order to reduce the feature distance. If the distance among them is higher than average, this indicates that these important key poses do not match well together and therefore the final distance is increased. For ambiguous key poses, that is, key poses with low discriminative value, pairings are not as important for the distance between sequences. On the other hand, a pairing of a discriminative and an ambiguous key pose should be disfavored as these key poses should match with instances with similar weights. Otherwise, the operator is based on the sign of dev(*i*, *j*), which means that low feature distances are favored (*z* = −1) and high feature distances are penalized (*z* = +1). This way, not only the shape-based similarity between key poses but also the relevance of the specific match is taken into account in sequence matching.

Once the nearest neighbor sequence of key poses is found, its label is retrieved. This is done for all the views that are available during the recognition stage. The label of the match with the lowest distance is chosen as the final result of the recognition; that is, the result is based on the* best view*. This means that only a single view is required in order to perform the recognition, even though better viewing angles may improve the result. Note that this process is similar to decision-level fusion, but, in this case, recognition relies on the same multiview learning model, that is, the bag of key poses.

## 6. Experimental Results

In this section, the presented method is tested on three datasets which serve as benchmarks. On this single- and multiview data, our learning algorithm is used with the proposed feature, and the results of the three chosen summary representations (variance, max value, and range) are compared. In addition, the distance-signal feature from Dedeoğlu et al. [10] and the silhouette-based feature from Boulgouris et al. [9], which have been summarized in Section 2, are used as a reference so as to make a comparison between features possible. Lastly, our approach is compared with the state of the art in terms of recognition rates and speed.

### 6.1. Benchmarks

The Weizmann dataset [7] is very popular in the field of human action recognition. It includes video sequences from nine actors performing ten different actions outdoors (*bending, jumping jack*,* jumping forward*,* jumping in place*,* running*,* galloping sideways*,* skipping*,* walking*,* waving one hand,* and* waving two hands*) and has been recorded with a static front-side camera providing RGB images of a resolution of 180 × 144 px. We use the supplied binary silhouettes without postalignment. These silhouettes have been obtained automatically through background subtraction techniques; therefore, they present noise and incompleteness. It is worth to mention that we do include the skip action, which is excluded in several other works because it usually has a negative impact on the overall recognition accuracy.

The MuHAVi dataset [8] targets multiview human action recognition, since it includes 17 different actions recorded from eight views with a resolution of 720 × 576 px. MuHAVi-MAS provides manually annotated silhouettes for a subset of two views from 14 (MuHAVi-14:* CollapseLeft, CollapseRight*,* GuardToKick*,* GuardToPunch*,* KickRight*,* PunchRight*,* RunLeftToRight*,* RunRightToLeft*,* StandupLeft*,* StandupRight*,* TurnBackLeft, TurnBackRight*,* WalkLeftToRight,* and* WalkRightToLeft*) or 8 (MuHAVi-8:* Collapse*,* Guard*,* KickRight*,* PunchRight*,* Run*,* Standup*,* TurnBack,* and* Walk*) actions performed by two actors.

Finally, our self-recorded DAI RGBD dataset has been acquired using a multiview setup of Microsoft Kinect devices. Two cameras have captured a front and a 135° backside view. This dataset includes 12 actions classes (*Bend*,* CarryBall*,* CheckWatch*,* Jump*,* PunchLeft*,* PunchRight, SitDown*,* StandingStill*,* Standup*,* WaveBoth*,* WaveLeft,* and* WaveRight*), performed by three different actors. Using depth-based segmentation, the silhouettes of the so-called* users* of a resolution of 320 × 240 px are obtained. In future works, we intend to expand this dataset with more subjects and samples and make it publicly available.

We chose two tests to be performed on these datasets as follows.

1. *Leave-one-sequence-out* cross validation (LOSO). The system is trained with all but one sequence which is used as test sequence. This procedure is repeated for all available sequences and the accuracy scores are averaged. In the case of multiview sequences, each video sequence is considered as the combination of its views.
2. *Leave-one-actor-out* cross validation (LOAO). This test verifies the robustness to actor-variance. In this sense, the sequences from all but one actor are used for training, while the sequences from the remaining actor, unknown to the system, are used for testing. This test is performed for each actor and the obtained accuracy scores are averaged.

### 6.2. Results

The feature from Boulgouris et al. [9], which has been originally designed for gait recognition, presents advantages regarding, for instance, robustness to segmentation errors, since it relies on the average distance to the centroid of all the silhouette points of each circular sector. Nevertheless, on the tested action recognition datasets, it returned low success rates, which are significantly outperformed by the other four contour-based approaches. Both the feature from Dedeoğlu et al. [10] and ours are based on the pointwise distances between the contour points and the centroid of the silhouette. Our proposal distinguishes itself in that a radial scheme is applied in order to spatially align contour parts. Further dimensionality reduction is also provided by summarizing each radial bin in a single characteristic value.  Table 2 shows the performance we obtained by applying this existing feature to our learning algorithm. Whereas on the Weizmann dataset the results are significantly behind the state of the art and the rates obtained on the DAI RGBD dataset are rather low, the results for the MuHAVi dataset are promising. The difference of performance can be explained with the different qualities of the binary silhouettes. The silhouettes from the MuHAVi-MAS subset have been manually annotated in order to separate the problem of silhouette-based human action recognition from the difficulties which arise from the silhouette extraction task. This stands in contrast to the other datasets whose silhouettes have been obtained automatically, respectively, through background subtraction or depth-based segmentation, presenting therefore segmentation errors. This leads us to the conclusion that the visual feature from [10] is strongly dependant on the quality of the silhouettes.

Table 2 also shows the results that have been obtained with the different summary functions from our proposal. The* variance* summary representation, which only encodes the local dispersion but not reflects the actual distance to the centroid, achieves an improvement in some tests at the cost of obtaining poor results on the MuHAVi actor-invariance tests (LOAO) and the DAI RGBD dataset. The* max value* summary representation solves this problem and returns acceptable rates for all tests. Finally, with *f*_range_, the range summary representation obtains the best overall recognition rates, achieving our highest rates for the Weizmann dataset, the MuHAVi LOSO tests, and the DAI RGBD dataset.

In conclusion, the proposed radial silhouette-based feature not only achieves to substantially improve the results obtained with similar features as [9, 10] but its low-dimensionality also offers an additional advantage in computational cost (feature size is reduced from ~300 points in [10] to ~20 radial bins in our approach).

### 6.3. Parameterization

The presented method uses two parameters which are not given by the constraints of the dataset and the action classes which have to be recognized and therefore have to be established by design. The first one is found at the feature extraction stage, that is, the number of radial bins *S*. A lower value of *S* leads to a lower dimensionality which reduces the computational cost and may also improve noise filtering, but, at the same time, it will reduce the amount of characteristic data. This data is needed in order to differentiate action classes. The second parameter is the number of key poses per action class and view *K*. In this case, the appropriate amount of representatives needs to be found to capture the most relevant characteristics of the sample distribution in the feature space, discarding outlier and nonrelevant areas. Again, higher values will lead to an increase of the computational cost of the classification. Therefore, a compromise needs to be reached between classification time and accuracy.

**Figure 4:**
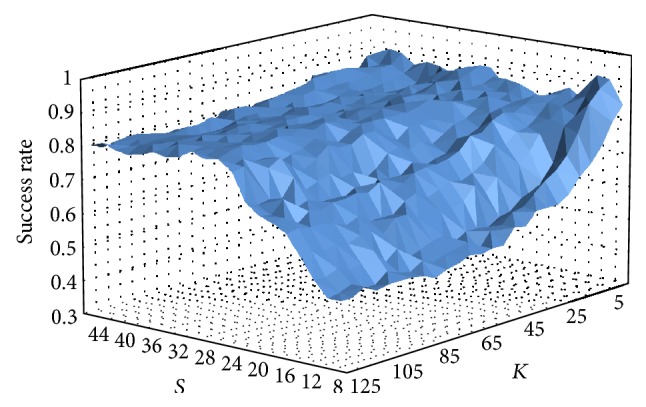
Median value of the obtained success rates for *K* ∈ {5,130} and *S* ∈ {8,46} (MuHAVi-8 LOAO test). Note that outlier values above or below 1.5 × IQR are not predominant.

In order to analyse the behavior of the proposed algorithm with respect to these two parameters, a statistic analysis has been performed. Due to the nondeterministic behavior of the* K-*means algorithm, classification rates vary among executions. We executed ten repetitions of each test (MuHAVi-8 LOAO cross validation) and obtained the median value (see Figure 4). It can be observed that a high value of key poses, that is, feature space representatives, only leads to a good classification rate if the feature dimensionality is not too low; otherwise, a few key poses are enough to capture the relevant areas of the feature space. Note also that a higher feature dimensionality does not necessarily require a higher number of key poses, since it does not imply a broader sample distribution of the feature space. Finally, with the purpose of obtaining high and reproducible results, the parameter values have been chosen based on the highest median success rate (92.6%), which has been obtained with *S* = 12 and *K* = 5 in this case. Since lower values are preferred for both parameters, the lowest parameter values are used if several combinations reach the same median success rate.

### 6.4. Comparison with the State of the Art

Comparison between different approaches can be difficult due to the diverse goals human action recognition methods may pursue, the different types of input data, and the chosen evaluation methods. In our case, multiview human action recognition is aimed at an indoor scenario related to AAL services. Therefore, the system is required to perform in real time as other services will rely on the action recognition output. A comparison of the obtained classification and recognition speed rates for the publicly available Weizmann and MuHAVi-MAS datasets is provided in this section.

The presented approach has been implemented with the.NET Framework using the OpenCV library [46]. Performance has been tested on a standard PC with an Intel Core 2 Duo CPU at 3 GHz and 4 GB of RAM with Windows 7 64-bit. All tests have been performed using binary silhouette images as input, and no further hardware optimizations have been performed.

Table 3 compares our approach with the state of the art. It can be seen that while perfect recognition has been achieved for the Weizmann dataset, our method places itself well in terms of both recognition accuracy and recognition speed when comparing it to methods that target fast human action recognition.

On the MuHAVi-14 and MuHAVi-8 datasets, our approach achieves to significantly outperform the known recognition rates of the state of the art (see Tables 4 and 5). To the best of our knowledge, this is the first work to report a perfect recognition on the MuHAVi-8 dataset performing the* leave-one-sequence-out* cross validation test. The equivalent test on the MuHAVi-14 dataset returned an improvement of 9.6% in comparison with the work from Cheema et al. [15], which also shows real-time suitability. Furthermore, our approach presents very high robustness to actor-variance as the* leave-one-actor-out* cross validation tests show, and it achieves to perform at over 90 FPS with the higher resolution images from the MuHAVi dataset. It is also worth mentioning that the training stage of the presented approach runs at similar rates between 92 and 221 FPS.

With these results, proficiency has been shown in handling both low and high quality silhouettes. It is known that silhouette extraction with admissible quality can be performed in real time through background subtraction techniques [47, 48]. Furthermore, recent advances in depth sensors make it possible to obtain human poses of substantial higher quality by means of real-time depth based segmentation [2]. In addition, depth, infrared, or laser sensors allow preserving privacy as RGB information is not essential for silhouette-based human action recognition.

## 7. Conclusion

In this work, a low-dimensional radial silhouette-based feature has been proposed, which in combination with a simple, yet effective, multiview learning approach based on a bag of key poses and sequence matching shows to be a very robust and efficient technique for human action recognition in real time. By means of a radial scheme, contour parts are spatially aligned, and, through the summary function, dimensionality is drastically reduced. This proposal achieves to significantly improve recognition accuracy and speed and is proficient with both single- and multiview scenarios. In comparison with the state of the art, our approach presents high results on the Weizmann dataset and, to the best of our knowledge, the best rates achieved so far on the MuHAVi dataset. Real-time suitability is confirmed, since performance tests returned results clearly above video frequency.

Future works include finding an optimal summary representation or the appropriate combination of summary representations based on a multiclassifier system. Tests with a greater number of visual sensors need to be performed so as to see how many views can be handled by the learning approach based on model fusion and to which limit multiview data improves the recognition. For this purpose, multiview datasets such as IXMAS [26] and i3DPost [49] can be employed. The proposed approach does not require that each viewing angle matches with a specific orientation of the subject because different orientations can be modelled if seen at the training stage. Nevertheless, since the method is not explicitly addressing view-invariance, it cannot deal with cross-view scenarios.

## Figures and Tables

**Algorithm 1:**
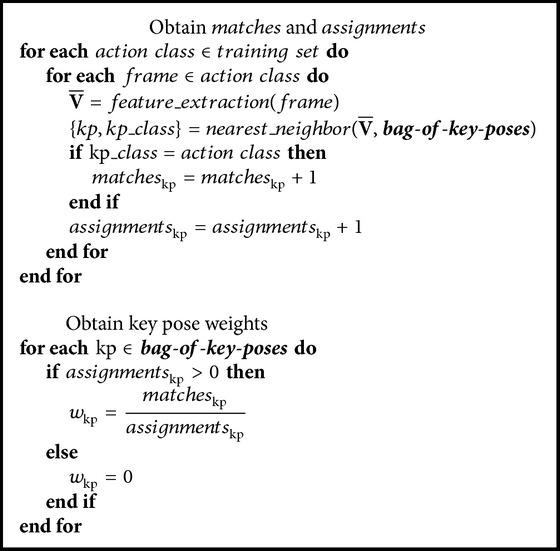
Pseudocode for obtaining the key pose weights *w*.

**Table 1:** Value of *z* based on the pairing of key poses and the signed deviation. *Ambiguous* stands for *w* < 0.1 and *discriminative *stands for *w* > 0.9. (These values have been chosen empirically.)

Signed deviation	Pairing	*z*
dev(*i*, *j*) < 0	*Discriminative *	−1
dev(*i*, *j*) > 0	*Discriminative *	+1
Any	*Ambiguous *	−1
Any	*Discriminative and ambiguous *	+1

**Table 2:** Comparison of recognition results with different summary values (*variance*, *max value*, and *range*) and the features from Boulgouris et al. [9] and Dedeoğlu et al. [10]. Best results have been obtained with *K* ∈ {5,130} and *S* ∈ {8,46}. (Bold indicates highest success rate.)

Dataset	Test	[9]	[10]	*f*_var_	*f*_max⁡_	*f*_range_
Weizmann	LOSO	65.6%	78.5%	90.3%	**93.5%**	**93.5%**
Weizmann	LOAO	78.5%	80.6%	92.5%	94.6%	**95.7%**
MuHAVi-14	LOSO	61.8%	94.1%	**95.6%**	91.2%	**95.6%**
MuHAVi-14	LOAO	52.9%	86.8%	70.6%	**91.2%**	88.2%
MuHAVi-8	LOSO	69.1%	98.5%	**100%**	**100%**	**100%**
MuHAVi-8	LOAO	67.6%	95.6%	83.8%	**98.5%**	97.1%
DAI RGBD	LOSO	50.0%	55.6%	50.0%	52.8%	**69.4%**
DAI RGBD	LOAO	55.6%	61.1%	52.8%	69.4%	**75.0%**

**Table 3:** Comparison of recognition rates and speeds obtained on the Weizmann dataset with other state-of-the-art approaches.

Approach	Number of actions	Test	Rate	FPS
$\dot{\text{I}}$kizler and Duygulu [11]	9	LOSO	100%	N/A
Tran and Sorokin [12]	10	LOSO	100%	N/A
Fathi and Mori [13]	10	LOSO	100%	N/A

Hernández et al. [14]^a^	10	LOAO	90.3%	98
Cheema et al. [15]	9	LOSO	91.6%	56
Chaaraoui et al. [5]	9	LOSO	92.8%	124
Sadek et al. [16]^a^	10	LOAO	97.8%	18

Our approach	10	LOSO	93.5%	263
Our approach	10	LOAO	95.7%	263
Our approach^a^	10	LOAO	97.8%	263

^a^Using 90 out of 93 sequences (repeated samples are excluded).

**Table 4:** Comparison of recognition rates and speeds obtained on the MuHAVi-14 dataset with other state-of-the-art approaches.

Approach	LOSO	LOAO	FPS
Singh et al. [8]	82.4%	61.8%	N/A
Eweiwi et al. [17]	91.9%	77.9%	N/A

Cheema et al. [15]	86.0%	73.5%	56
Chaaraoui et al. [5]	91.2%	82.4%	72
Chaaraoui et al. [6]	94.1%	86.8%	51

Our approach	**95.6%**	**88.2%**	**93**

**Table 5:** Comparison of recognition rates and speeds obtained on the MuHAVi-8 dataset with other state-of-the-art approaches.

Approach	LOSO	LOAO	FPS
Singh et al. [8]	97.8%	76.4%	N/A
Martínez-Contreras et al. [18]	98.4%	—	N/A
Eweiwi et al. [17]	98.5%	85.3%	N/A

Cheema et al. [15]	95.6%	83.1%	56
Chaaraoui et al. [5]	97.1%	88.2%	81
Chaaraoui et al. [6]	98.5%	95.6%	66

Our approach	**100%**	**97.1%**	**94**
